# IgG N-glycosylation in autoimmune diseases

**DOI:** 10.3389/fimmu.2026.1933960

**Published:** 2026-10-05

**Authors:** Caroline Pumpe, Sonja Vermeren

**Affiliations:** Centre for Inflammation Research, Institute for Regeneration and Repair, The University of Edinburgh, Edinburgh, Scotland, United Kingdom

**Keywords:** agalactosylation, autoimmune disease, IgG antibody glycosylation, IgG N-glycan, immunomodulation, FcγR

## Abstract

IgG is the most abundant antibody in the human circulation. It carries a single conserved glycosylation site, Asparagine 297, in its Fc domain. This N-glycan is critical for the ability of the antibody to activate immune cells via binding to, and cross-linking Fc receptors in order to elicit functional responses. First documented in the context of rheumatoid arthritis (RA), the IgG N-glycan may undergo changes that are correlated to host health. We present a narrative in-depth comparison of changes incurred by IgG N-glycans in autoimmune diseases; RA, systemic lupus erythematosus, multiple sclerosis, type I diabetes, primary biliary cholangitis and inflammatory bowel disease (Crohn’s disease and ulcerative colitis). Based on a systematic literature search, we identify common traits and features that are specific for any one autoimmune disease. Our analysis shows that the single most common trend in most of the conditions analyzed here is increased IgG agalactosylation. Changes to the IgG N-glycan typically accompany and often precede disease onset and flare-ups, while they decrease in remission and pregnancy. The correlation of agalactosylated IgG with inflammatory disease is so striking that the agalactosylated IgG N-glycan has long been viewed as inflammatory and even pathogenic. Yet, despite the association with inflammation, any mechanism by which disease-associated IgG N-glycans may mediate their pro-inflammatory nature remains enigmatic.

## Introduction

Antibodies form a critical part of the immune system, binding to and opsonizing undesirable targets, thereby marking them for destruction, via antibody- and/or complement dependent cytotoxicity (ADCC/CDCC) or antibody-dependent phagocytosis. Several antibody classes are known, of which, in humans, IgG is best characterized. Four IgG subclasses exist, IgG1-4. Of these, IgG1 is best understood and most abundant in the circulation (60-70% in plasma) followed by the lesser understood IgG2 (20-30%), and the comparatively rare IgG3 (5-8%) and IgG4 (1-3%) species (1). Mouse antibodies perform the same functions as their human counterparts, with mouse IgG2a/b being the homologue of human IgG1. Importantly, a number of species-specific differences exist between mouse and human antibodies and their receptors, which are reviewed in depth elsewhere (2, 3). In the interest of clarity, this article will concentrate on human antibodies.

Made up of two heavy and two light chains that are joined by disulfide bridges, the variable region of the IgG Fab domain determines its antigen binding specificity (**Figure 1A**). Despite being highly conserved and following the same blueprint, some structural variations exist between IgG subclass constant regions, with differences localized in particular to the hinge region. Hence IgG1, 2 and -4 have short hinge regions, whereas IgG3 has an extended hinge region that is sensitive to proteolytic cleavage and is distinguished by higher numbers of inter-heavy chain disulfide bonds.

**Figure 1:**
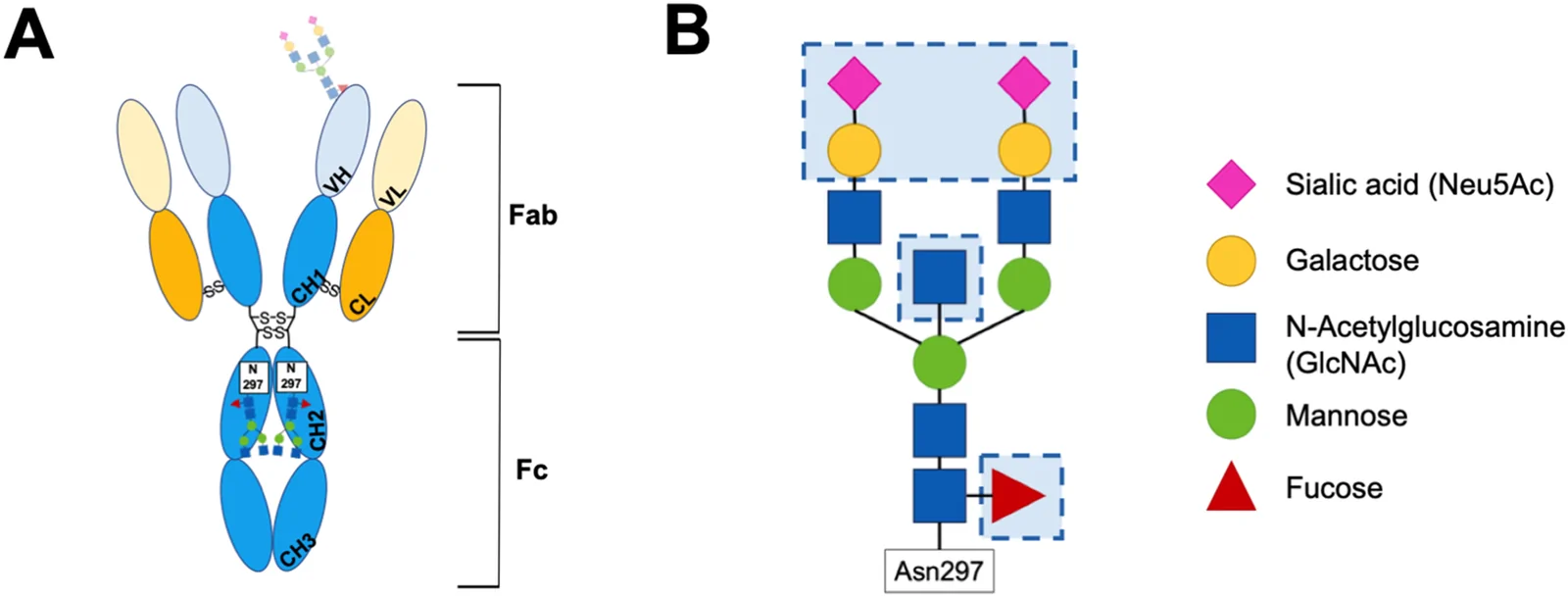
Schematic of IgG glycans. **(A)** IgG is made up of two heavy and two light chains, which are covalently linked by disulfide bridges. The heavy chains are also linked to one another by disulfide bridges that localise to the hinge region which separates the variable from the constant domains of the antibody. IgG1, the example shown here has a short hinge region with two inter-heavy chain disulfide bridges. The IgG Fc domain contains a single conserved N-glycan linked to the invariable Asn 297. In addition, Fab glycans may occur in the variable domain. This is more common in some autoantibodies, such as anti-citrullinated protein antibodies in RA. **(B)** The invariable IgG N-glycan consists of a conserved backbone made up of GlcNAc (blue square) and mannose (green circle) residues, here shown with a white background. The glycans may feature an additional fucose (red triangle), galactose (yellow circle) and sialic acid (pink diamond) as well as a bisecting GlcNAc, here identified by their blue background. Note, fucose only occurs in the position shown here and sialic acid is always a terminal residue and bound to galactose as shown.

A unique functional feature of IgG4, lies in its inter-heavy chain bonds being unstable. This permits IgG4 to dissociate into half molecules that may subsequently re-associate in a process called Fab-arm exchange to form bispecific antibodies that are functionally monovalent (4, 5).

Differences in the Fc domains of the IgG subclasses confer differential affinities of IgG subclasses to the classical Fcγ receptors, FcγRI (CD64), FcγRIIa (CD32a), FcγRIIb (Cd32b), FcγRIIIa (CD16a) and FcγRIIIb (CD16b) (6). With differential expression of the FcγRs by various immune cells this results in divergent intracellular signaling and ultimately distinct immune responses, shaping immunity and inflammation alike.

The neonatal Fc receptor, FcRn functions as IgG transporter and regulates IgG half-life: it binds monomeric IgG1, 2 and 4 efficiently in the acidic conditions of the endosome, protecting them from lysosomal degradation which explains their long half-lives of ~3 weeks. Owing to its extended hinge region IgG3 exhibits poorer binding to FcRn and less protection from degradation, resulting in the shorter half-life of only several days (7, 8).

Structural differences between IgG subclasses are also the reason for different complement activation abilities of the IgG subclasses (IgG3 > IgG1 > IgG2 > IgG4). C1q binding is the first step in the activation of complement. Not only are there differences in IgG subclass affinities for the gC1q headpiece (9), but CDCC can only be triggered by antibody oligomers (upwards of tetramers). Relatedly, complement binding was also shown to be correlated with oligomerization of antigen-bound IgG, and can be maximized by mutations that enhance IgG oligomerization. This may explain why IgG4, which forms at best small immune complexes is virtually unable to activate complement (10–12).

## Antibody glycosylation

Just like other proteins, antibodies are subject to post-translational modifications. The perhaps best known of these is antibody glycosylation. Glycosylation involves the attachment of carbohydrate moieties, so-called glycans, to secreted proteins on specific amino acid residues. Two types of protein glycosylations are distinguished, N-linked and O-linked. N-glycosylation involves the attachment of an N-acetylglucosamine (GlcNAc) to an asparagine residue in an Asn-X-Ser/Thr motif, whereas in O-linked glycosylation N-acetylgalactosamine (GalNAc) is attached to a serine or threonine residue of an antibody.

All human IgGs contain the conserved N-linked glycosylation site, Asn 297, which is generally occupied. IgG3 is the only human IgG antibody to contain an additional N-linked glycosylation site in its constant regions, Asn 392, which has an occupancy of ~12%. IgG3 moreover contains three O-linked glycosylation sites in its enlarged hinge region that have partial occupancy (~10%). The significance of O-linked IgG3 glycosylations remains largely unexplored (13, 14).

Given that it holds critical roles for Fc conformation, stability, FcγR binding, and effector functions, all of which are affected in deglycosylated antibodies (15–17), the N-glycan linked to IgG Asn 297 has been investigated in much depth. It consists of a conserved backbone made up of two N-acetylglucosamine (GlcNAc) and three mannose residues, as well as two further GlcNAc residues, forming a tree. It may also feature additional galactose, terminal N-acetylneuraminic acid (Neu5a) residues, fucose and additional bisecting GlcNAc residues. Neu5a is the only sialic acid that is synthesized *de novo* by human cells, so for simplicity’s sake we will refer to it from here on as sialic acid. The final N-glycan structure conforms to a strict pattern with several possible variations (**Figure 1B**). At any time, a mixture of several different N-glycan variations co-exist on an individual’s circulating IgG molecules.

Just like IgG and their receptors, IgG N-glycosylation varies between species. Mice are frequently used as experimental models, but unlike humans, mouse cells can synthesize both Neu5a and N-glycolylneuraminic acid (Neu5g) (18). Mouse IgG N-glycans may moreover take conformations that are not found in human; for example, they may contain α1,3-galactosylation, whereas human N-glycans only contain  β-glycosidic linkages, rendering mouse N-glycans immunogenic in humans (19).

Unlike a protein’s amino acid sequence, its protein glycosylation is not genetically encoded. Rather, glycosylation of particular residues is thought to be regulated in an epigenetic fashion (20). IgG Fc N-glycans are added intracellularly in a complicated, glycosyltransferase- and glycosidase-dependent fashion. First, a mannose-rich glycan precursor, Glc3Man9GlcNAc2 is transferred onto Asn297 in the endoplasmic reticulum (ER) by oligopeptide oligosaccharide transferase I. Subsequent N-glycan remodeling involves the removal of glucose and mannose residues by ER and Golgi-localized glucosidases I and II, and α-1,2-mannosidases, respectively. The resulting Man5GlcNAc2 structures are further remodeled by mannosidase II, which removes two terminal mannose residues. This creates sites for GlcNAc addition by GlcNAc transferase II, forming the characteristic GlcNAc2Man3GlcNAc2 core of the glycan tree. (**Figure 1B**). This is followed by subsequent additional post-translational processing steps in the Golgi apparatus until the final complex N-glycan emerges (21). Major glycosyltransferases that are involved in the modifications of IgG antibody N-glycans prior to their secretion from B cells and their function are listed in **Table 1**.

**Table 1:** Major glycosyltransferases involved in IgG antibody N-glycan remodeling.

Gene	Protein	Function in IgG N-glycan generation
*B4GALT1*	β-1,4-galactosyltransferase	Transfers galactose to terminal GlcNAc residues in β-1,4-linkage on complex N-linked glycans on IgG.
*FUT8*	α-1,6-fucosyltransferase 8	Responsible for α-1,6-linked fucosylation of the IgG N-glycan core.
*ST6GAL1*	β-galactoside α-2,6-sialyl-transferase 1	Responsible for α-2,6 sialylation of terminal galactose on IgG Fc N-glycans.
*MTGAT3*	β-1,4-N-acetylglucos-aminyltransferase III	Transfers unique bisecting GlcNAc branch into IgG N-glycans

In addition to intracellular glycan processing, there is also evidence that glycans may be modified extracellularly once the IgG molecule has been secreted. This is particularly true for sialylation, a modification that B cells are not thought to catalyse very efficiently (22–25). For example, extracellular sialylation was observed to be mediated by circulating glycosyltransferases secreted by hepatocytes and incorporating platelet-derived sialic acid (22).

In addition to the invariable Fc-localised N-glycans, antibodies may also contain additional domain (Fab) glycosylation, which introduces an additional level of variability to their antigen binding sites. Interestingly, Fab glycosylation has been linked to autoimmunity, where Fab glycans are thought to be introduced during somatic hypermutation (26, 27). However, Fab glycans do not necessarily share characteristic structural features of Fc glycans.

## Characteristically changed IgG N-glycans in autoimmune disease

Antibody Fc N-glycans are changeable. Characteristic changes to IgG N-glycans are associated with disease progression and severity in autoimmune diseases. An association was first observed with RA and has since been observed also in a range of other immune diseases. IgG N-glycosylation was proposed as a potential biomarker (28), and it was hypothesized that each condition may carry a unique N-glycan profile (29).

In the light of the large body of literature investigating IgG glycosylation in many autoimmune diseases, we performed a systematic literature search to identify N-glycan characteristics in a selection of autoantibody- and non-autoantibody-mediated diseases, identifying a total of 103 articles for inclusion into our analysis (**Supplementary Figure S1** for details). Most were observational studies that compared IgG glycosylation in patients and matched healthy controls, while others compared the effects of medication and pregnancy on IgG glycosylation.

The following diseases were included into the literature search:

- **Rheumatoid arthritis** (RA), a systemic autoimmune disease primarily affecting synovial joints that is characterized by the presence of autoantibodies, notably anti-citrullinated protein antibodies (ACPA).
- **Systematic lupus erythematosus** (SLE), a multisystem autoimmune disease with a broad range of symptoms that is characterized by autoantibodies recognizing cellular components, e.g. anti-nuclear antibodies (ANA).
- **Multiple sclerosis** (MS), a chronic demyelinating disease of the central nervous system. A diagnostic feature of MS is the presence of oligoclonal bands, oligoclonal antibodies of elusive specificity, in the cerebrospinal fluid.
- **Type 1 diabetes mellitus** (T1D) an autoimmune disease in which insulin-generating beta cells of the pancreas are destroyed by the immune system that is accepted as T cell-mediated, although there are characteristic anti-islet antibodies.
- **Primary biliary cholangitis** (PBC), an autoimmune disease affecting the liver, that is characterized by autoantibodies, notably antimitochondrial antibodies.
- **Inflammatory bowel disease** (IBD), specifically, Crohn’s disease (CD) and ulcerative colitis (UC), that are immune-mediated/autoimmune and involve loss of tolerance against elements of the gut microbiome. These conditions are characterized by anti-microbial antibodies, e.g. anti-Saccharomyces cerevisiae; anti outer-membrane protein of *E.coli* and/or autoantibodies, e.g. anti-perinuclear neutrophil cytoplasmic antibodies, respectively.

**Table 2** summarizes the IgG glycan characteristics obtained from carefully analyzing the papers included into this study by autoimmune disease. The articles employed various methods to analyze glycans. Earlier studies mostly included comparatively simplistic, lectin-based analyses. Such lectin-based assays characterized glycan residues present on IgG isolated from serum samples, providing an overview based on binding specificities of particular lectins (**Figure 2** for some examples). Due to the comparative ease of using lectins, without any need for sophisticated specialist equipment, lectin-based analyses have remained popular to this day. Alongside this, increasingly sophisticated high-performance liquid chromatography and mass spectrometry-based approaches were employed from ~2010s. These approaches are able to provide qualitative and quantitative information on IgG N-glycan traits and their structures even from small amounts of starting material. This permitted the inclusion of additional preparative steps prior to performing the structural analysis of glycans, in combination with which it is possible to perform site-specific (Fc or Fab) and/or IgG subclass analyses.

**Table 2:** Autoimmune disease-associated IgG N-glycans share common traits.

Condition	IgG glycan characteristics	
Rheumatoid Arthritis	**↑ Agalactosylation** (28, 30–44) **↓ Galactosylation** (32, 42, 45–60) **↓ Sialylation** (40, 42, 54, 55, 57, 58, 61, 62) ↑ Bisecting GlcNAc (28, 42, 44, 57–79) ↑ Fucose (52, 55, 58, 63–65)	
Systemic Lupus Erythematosus	**↑ Agalactosylation** (31, 66, 67) **↓ Galactosylation** (32, 48, 48, 51, 51, 66–68) **↓ Sialylation** (40, 63, 66, 69, 70) **↑ Bisecting GlcNAc** (66, 67) ↑/↓ Fucose (66, 67, 71–73)	
Multiple Sclerosis	**Serum**	**Cerebrospinal Fluid**
	↑/– Galactosylation (74–77) ↑ Sialylation (76–78) ↓ Fucose (78)	**↑ Agalactosylation** (74, 79) **↑ Monogalactosylation** (75) ↓ Digalactosylation (75) **↓ Sialylation** (75, 76, 79) ↑ **Bisecting GlcNAc** (75, 79) ↑ Fucose (75, 79)
Type 1 Diabetes	↑ Galactosylation (80, 81) ↑ **Sialylation** (80–82) ↑ **Bisecting GlcNAc** (80–82)	
Primary Biliary Cholangitis	↑ **Agalactosylation** (83, 84) ↓ **Galactosylation** (83, 85) ↓ **Sialylation** (85, 86) ↑ Bisecting GlcNAc (85) ↑/↓ Fucose (subclass dependent) (83) ↑ **N-glycan branching** (83)	
Crohn’s Disease	↑ **Agalactosylation** (87–90) ↓ **Galactosylation** (48, 50, 51, 88, 89, 91) ↓ **Sialylation** (88, 89, 91) ↑/↓/– Bisecting GlcNAc (subclass dependent) (88, 89)	
Ulcerative Colitis	↑ **Agalactosylation** (87–89) ↓ **Galactosylation** (88, 89, 91) ↓ **Sialylation** (91) ↓/– Bisecting GlcNAc (subclass dependent) (88, 89) ↓ Fucose (88, 89)	

All comparisons relate to matched healthy control samples analyzed at the same time as the patient samples. ↑, Higher than healthy controls; ↓, Higher than healthy controls; –, No differences to healthy controls; Major traits are in bold. See **Supplementary Figure S1** for numbers of articles in each category, inclusion and exclusion criteria etc.

Information relating to disease-associated glycans extracted from included articles is summarized in this table.

**Figure 2:**
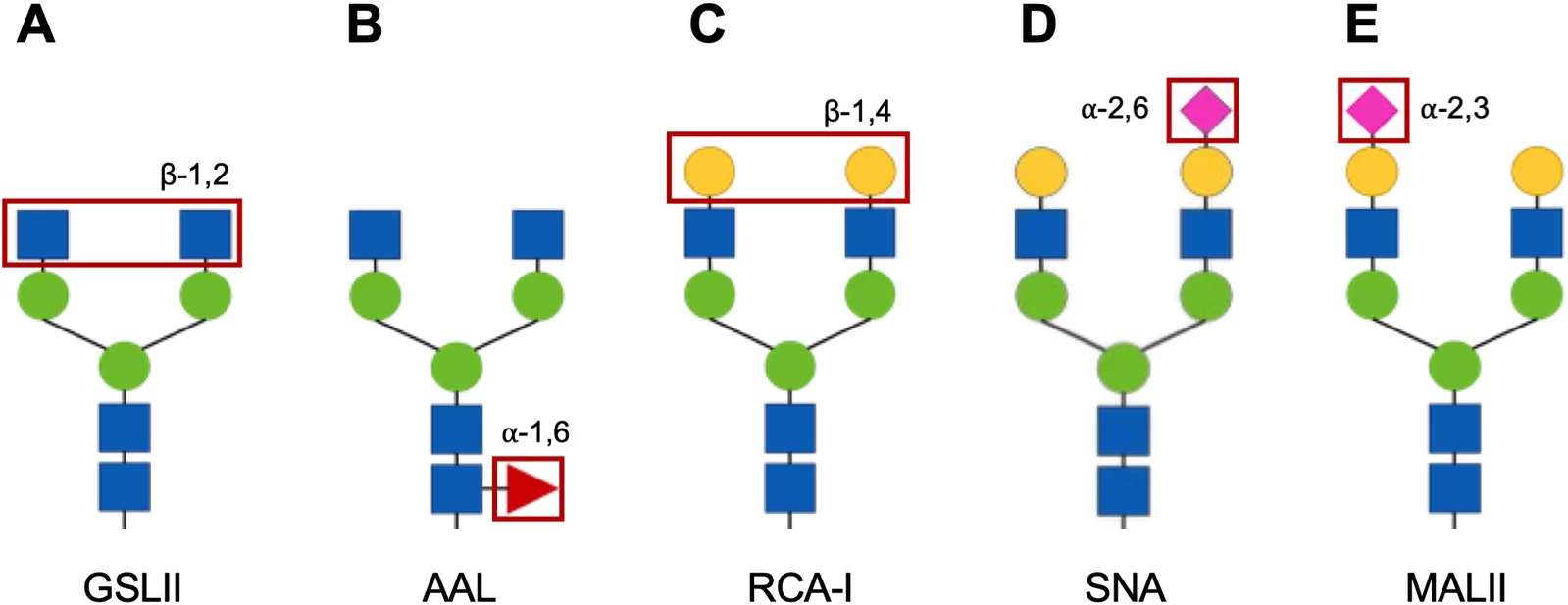
Glycan structures recognized by some commonly used lectins. **(A)** Griffonia (Bandeiraea) simplicifolia Lectin II (GSLII, BSLII) recognizes terminal α- and β-linked GlcNAc residues on oligosaccharides and be used to identify agalactosylation. **(B)** Aleuria aurantia lectin (AAL) recognizes various fucose linkages to GlcNAc (α-1,2; α-1,3; α-1,4; α-1,6) and can be used to identify fucosylation. **(C)** Ricinus Communis agglutinin I (RCA-I) recognizes terminal β-linked galactose and can be used to identify galactosylation. **(D)** Sambucus nigra lectin (SNA) recognizes α-2,6-linked sialic acid, and can be used to recognize sialylated glycans (human IgG). **(E)** Maackia amurensis lectin II (MALII) recognizes α-2,3-linked sialic acid, which could be incorporated into IgG produced in CHO cells or by mouse cells (18). Numbers refer to the carbons involved while α- and β- relate to the stereochemistry of the glycosidic bond in question.

Different methods were employed to statistically analyze the results obtained in these studies. While trends, encouragingly, remained steady throughout the changing analysis methodology employed, it was therefore unfortunately not possible to integrate the results of these disparate studies in a quantitative fashion. Similarly, due to a lack of standardized reporting, differences in N-glycan structure obtained from HPLC/MS-based methods could not be meaningfully compared with those obtained by methods based on lectins, plant-derived proteins that bind to particular carbohydrate moieties in a specific context.

With different techniques employed to measure glycans, and not all features measured in all reports, galactosylation, agalatosylation and sialylation are listed separately. Agalactosylation refers to a glycan structure that is devoid of galactose, whereas decreased galactosylation does not necessarily mean increased agalactosylation, but may reflect a shift from digalactosylated to monogalactosylated glycans and/or changes in the overall distribution of galactose residues in the glycan pool. The use of lectins may inform on the presence of any carbohydrate only in a specific configuration, with a specific linkage etc. In contrast, a mass spectrometric peak will inform on the total mass of a glycan that may be compatible with more than one conformation or composition.

### Rheumatoid arthritis

The oldest, and also largest number of studies from many different laboratories analyzed IgG N-glycans from RA patients. Due to the age of many of the studies, RA is also the condition for which there are both studies that employed old-fashioned approaches with relatively small cohorts, while others used state-of-the-art analysis techniques on large cohorts of patients. Reassuringly, they all arrived at similar conclusions. Increased levels of agalactosylated core-fucosylated glycans (**Figure 3A**) were frequently reported (44, 56, 92), and found correlated with increased disease activity, disease progression and relapse (54, 55, 93). In RA inflammation is localized to the synovial joints. Agalactosylation was found to be most pronounced in synovial fluid IgG irrespective of antibody specificity by some studies (65, 92). Others reported that autoantibodies, anti-citrullinated protein antibodies (ACPA) and rheumatoid factor (RF) were even less galactosylated than total IgG, irrespective of location (94, 95). Indeed, one report noted RF binding to Fc increased with reduced galactosylation, and was associated with increased RF titres and RA disease activity (53, 95).

**Figure 3:**
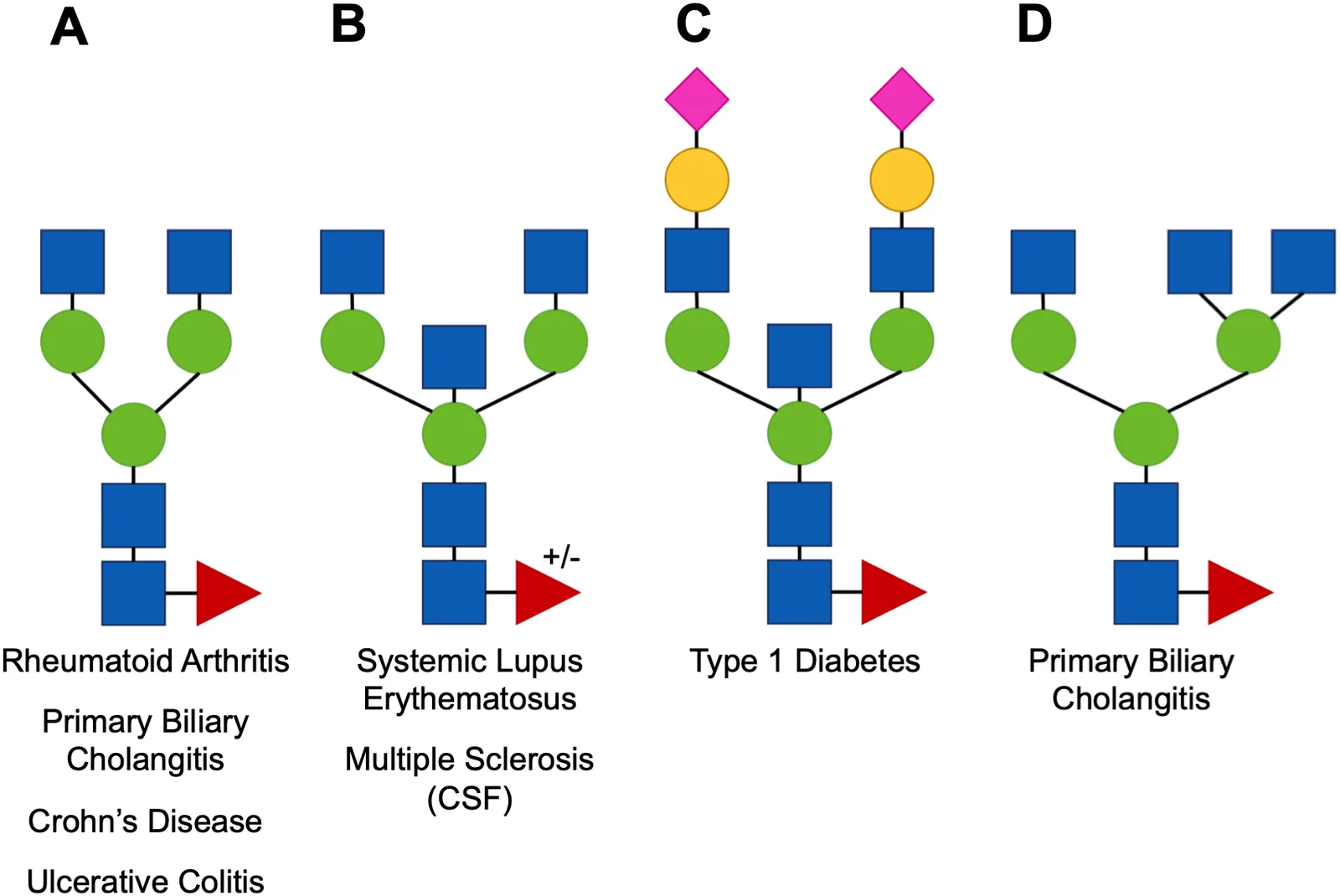
IgG N-glycans characteristic for autoimmune disease. **(A)** Increased biantennary agalactosylated IgG, also called FA2, was a characteristic trait of most autoimmune diseases discussed here and associated with increased disease severity. Some diseases showing additional trends: **(B)** Bisecting GlcNAc, also called A2B, in SLE; **(C)** hypersialylation and excessive branching (here shown with bisecting GlcNAc; structure also called FA2BG2S2) in T1D Fab glycans; **(D)** distinctive branching phenotype (structure shown here also called FA3) in PBC.

Several studies observed that fucosylation of the N-glycan in IgG from RA patients was similar to that of healthy controls (40, 57, 65). Others described a minor increase in IgG glycan fucosylation from RA patients compared to healthy controls (55, 92). Reports on the abundance of bisecting GlcNAcs were variable (46, 54, 57, 94). It is unclear what the underlying reasons are between these contradictions. It is conceivable that they might be due to different analytical techniques employed, or that there were differences in the cohorts, e.g. due to different genetic predispositions in the cohorts.

Several studies analyzed the changes to IgG glycans in response to treatment of RA patients. These studies included a range of different treatments, ranging from DMARDs [methotrexate, sulfasalazine (43, 55)] and glucocorticoids (64) to biologicals (anti-TNF, anti-IL6) alone or in combination with DMARDs (91, 96–99), with length of treatment ranging from 2 weeks to 12 months. Remarkably, all reports concurred: IgG N-glycan agalactosylation was reduced, while galactosylation and sialylation (where measured) were increased in response to treatment.

Increased IgG N-glycan galactosylation and sialylation were observed throughout pregnancy in healthy women. Interestingly, this also applied with pregnant RA patients, with all IgG subclasses affected. Increased IgG galactosylation during pregnancy was associated with disease remission. Following pregnancy, the IgG N-glycans of RA patients reverted to decreased galactosylation and sialylation; steeper declines were associated with pronounced early post-partum disease flares (54, 100–102).

### Systemic lupus erythematosus

Changes observed with IgG glycans of SLE patients shared several of the attributes of antibody glycans reported in RA. Both IgG sialylation and galactosylation were reduced compared with healthy controls; reduced galactosylation/sialylation was associated with increased disease severity (48, 66, 69), and this change was reported to be more pronounced in autoantibodies than total serum IgG (63, 73).

Contrasting with RA, SLE IgG was characterized by glycans carrying increased levels of bisecting GlcNAc (**Figure 3B**). Two large studies also reported reduced core fucosylation (66, 67), where lower core fucosylation was correlated with lupus nephritis and longer disease duration (66, 103). The most likely reason for this is steric hindrance, since bisecting GlcNAcs hinder IgG fucosylation by α-1,6-fucosyltransferase (104, 105).

While the effects of treatment and pregnancy on IgG N-glycans were not analyzed as extensively as in RA, treatment with biologicals and pregnancy were both reported to correlate with increased IgG galactosylation/sialylation (70, 91, 106), while lower IgG sialylation in pregnancy was correlated with higher disease activity and fetal loss.

### Multiple sclerosis

Differential IgG glycosylation profiles were observed in serum and cerebrospinal fluid (CSF), the location of the oligoclonal bands (OCBs) that occur in >90% of MS patients (107, 108). OCBs refers to (oligoclonal) IgG antibodies that are locally produced by clonal B cells, and that represent a diagnostic marker in MS. Serum IgG from people with MS was characterized by increased sialylation compared to that from healthy controls, with increased mono- and even disialylated IgG reported (76, 78). In contrast, CSF IgG N-glycans were characterized by increased agalactosylation, and decreased galactosylation and sialylation compared to serum IgG from MS patients and also healthy controls. Interestingly, decreases in CSF IgG galactosylation and sialylation were associated with increased intrathecal IgG production as well as higher CSF cell numbers, both signs of inflammation and damage (74, 75, 79). A very recent study showed that enzymatic glycan removal from CSF-derived IgG reduced OCB complexity and reduced neurotoxicity of CSF-derived immune complexes *in vitro* (77).

In relapsing-remitting MS, increased bisecting GlcNAc and fucosylation of CSF-derived IgG glycans was correlated with relapse. IgG glycan features characteristic for MS were however not correlated with disease duration or disability status (78, 79).

### Type I diabetes

T1D IgG glycan profiles were largely reported to be distinct from those of the other autoimmune diseases discussed as part of this study. T1D glycans were characterized by increased bisecting GlcNAc, as well as increased N-glycan galactosylation and sialylation, including disialylation (**Figure 3C**), when compared to those from healthy donors. These characteristics were observed in blood samples from large cohorts of children that had been obtained near disease onset (80, 81), and the changes were reported to persist into adulthood (82, 109).

Not entirely unexpectedly, more complex glycans, as well as increased galactosylation and sialylation were found to be associated with poor glycemic control and onset of kidney disease. The same study also unearthed an association between diabetic kidney disease and increased IgG glycan agalactosylation (82), reminiscent of the pattern observed in the other autoimmune diseases analyzed here.

Interestingly, disialylated Fc N-glycans were reported to be anti-inflammatory, certainly in the context of IVIG (intravenous immunoglobulin) and the mouse K/BxN model of RA (110, 111). In the wake of this exciting observation, antibody sialylation became the subject of intense research which continues to this day (see also below).

To which extent these observations are relevant to the observations with IgG glycans in T1D is unclear, since a recent study performed a separate analysis of Fab- and Fc glycans in a large cohort of T1D patients and matched controls (80). The results of this study suggested that the increased levels of galactosylation, mono- and disialylation of IgG in T1D were from abundant Fab but not Fc glycans. When comparing the two, the abundance of monogalactosylated glycans with bisecting GlcNAc was significantly different. In contrast, significant differences between Fc glycans of healthy controls and patients with T1D were not reported.

In summary, according to several studies T1D Fc N-glycans do not appear to share the reduced galactosylation that characterized the remaining autoimmune diseases discussed here, although there is a possibility that decreased galactosylation does occur as it was reported in the context of worsened diabetic kidney disease (82), and may be masked by the increased sialylation occurring under condition of poor glycemic control to Fc and/or Fab glycans.

### Primary biliary cholangitis

IgG1 N-glycans in PBC were characterized by increased agalactosylation, as well as decreased galactosylation and sialylation, while IgG3/4 were less fucosylated than those from healthy controls. The lower abundance of digalactosylated and monosialylated IgG1 N-glycans correlated with markers of liver disease (83, 85, 86). While these features fit with the profile also observed in other autoimmune diseases discussed here, a distinctive feature of PBC was increased N-glycan branching structures [**Figure 3D** (83)]. This glycan branching was associated with longer disease duration and liver cirrhosis, and distinguished PBC from primary sclerosing cholangitis, a rare, progressive non-autoimmune liver disease.

### Inflammatory bowel disease

While not classical autoimmune diseases, the characteristic antibodies for CD and UC shared with those of classical autoimmune diseases the N-glycan pattern of reduced galactosylation and sialylation (87–89, 91). These characteristics were observed in geographically and genetically distinct populations; they were associated with disease severity and the need for surgical intervention. Interestingly, any decrease in IgG glycan galactosylation observed was more intense in CD compared to UC (51, 87). In CD patients, characteristic changes could be observed up to 6 years prior to diagnosis (90). Upon treatment with biologicals, agalactosylated glycans did not revert in UC patients, whereas IgG galactosylation and sialylation did increase in CD patients within 6 months of treatment (91, 112).

## Associations suggestive of an inflammatory agalactosylated glycan conformation

When compiling the results of all these studies, obvious common trends emerged between IgG N-glycans in the majority of the diseases analyzed, RA, SLE, MS, PBC, UC and CD, while T1D appears to be a notable exception. Common core features of the ‘inflammatory glycan signature’ were, reduced galactosylation/increased agalactosylation, which coincides with concomitant decreases in sialylation. In contrast, other glycan features, e.g. relating to branching were observed in some but not all diseases. Taken together, this suggests that increased agalactosylation of IgG glycans is not specific for any one autoimmune disease, as had been speculated by some (28, 29). Rather, all these autoimmune diseases shared increased levels of IgG N-glycan agalactosylation, a glycoform that, due to this association with inflammation, has been referred to as ‘inflammatory’ or even ‘pathogenic’ (45, 70, 90, 113). The inflammatory glycan signature was observed by a large number of different studies with many large cohorts in genetically and geographically diverse populations. The results were moreover obtained with several of different technical approaches, further increasing confidence in the findings.

It stands to reason that an autoantibody that drives autoimmune disease may carry an inflammatory signature, but interestingly, many studies reported that glycan changes affect total circulating IgG as well as autoantibodies (**Table 1**). An important question therefore is: Do the glycan changes summarized above arise and promote inflammation, or do they in fact arise as a result of inflammation? To our knowledge this question remains unanswered at present.

## Effects of IgG glycosylation on FcγR binding

As previously discussed, human immune cells express a range of IgG receptors, the FcγRs. With the exception of FcγRI, they are low affinity receptors, and with the exception of FcγRIIb these are activating receptors. In the context of binding to immune complexes, which cross-link FcγRs, receptor binding is noticeably amplified (6).

Interactions of IgG Fc with the FcγR are dependent upon the presence of the IgG N-glycan. Monomeric aglycosylated IgG1 exhibited approximately 40-fold reduced binding to the high affinity receptor FcγRI, whereas immune complexes were less affected (17, 114). Showcasing the importance of the N-glycan, binding of aglycosylated IgG to low affinity FcγRs was negligible/abolished with monomeric IgG and even with immune complexes (15, 115). Similarly, minimal glycosylation of IgG with only the penultimate GlcNAc and its fucose residue exhibited dramatically reduced FcγR binding compared to IgG with longer glycans, for monomeric IgG, small and large immune complexes (15, 17).

Work by several laboratories has established that afucosylation promotes binding of IgG to human FcγRIIIa, reportedly up to ~20-fold, with a somewhat lesser effect reported for FcγRIIIb (116–118). This has clear repercussions on the biology of FcγRIIIa-expressing immune cells, NK cells, as well as macrophages and monocytes, with cytotoxicity and phagocytic efficiency significantly improved when targets are opsonized by afucosylated IgG antibodies. Literature related to this, and the mechanism by which afucosylation improves FcγRIIIa binding by IgG has been extensively reviewed elsewhere (119).

Contrasting with fucosylation, which is at best a minor change in most autoimmune diseases (**Table 1**), galactosylation makes only a minor contribution to FcγR binding in the context of monomeric IgG (118, 120, 121), although some studies suggested 2 galactose residues to mediate improved binding to FcγRIIa/b and FcγRIIIa/b over those containing no or one galactose residue (115, 122). Interestingly, immune complexes generated from galactosylated IgG bound significantly stronger than agalactosylated IgG to any FcγRs we tested (123). NMR studies and computational modeling suggest that the presence of galactose in the glycan stabilizes the IgG Fc region, offering a possible reason for the observation (124).

Reports on effects of IgG antibody glycan sialylation on FcγR binding are somewhat contradictory, with improved binding to FcγRIIa/b and FcγRIIIa/b reported by some (122, 125), and reduced binding to the same receptors reported by others (110, 126). Of note, antibody sialylation was anti-inflammatory in the context of IVIG in a range of *in vivo* models of sterile inflammation, a subject that has been reviewed in depth elsewhere (127). Interestingly, these effects are not thought to be mediated by altered binding efficiency to classical FcγRs. Rather, anti-inflammatory functions of sialylated IgG N-glycans were proposed to be mediated by upregulation of the inhibitory FcγRIIb, resulting in inhibitory signaling. In addition, in somewhat controversial claims, sialylated IgGs were reported to be mediated by so-called type 2 Fc receptors, including C-type lectins DC-SIGN and CD23. These were proposed to induce anti-inflammatory pathways that result in the release of anti-inflammatory cytokines, as well as B cell apoptosis, reducing inflammation and auto-immunity via mechanisms that were reviewed in detail elsewhere (128, 129).

## Effects of IgG glycosylation on complement deposition

IgG glycosylation also regulates complement deposition and ultimately formation of the membrane attack complex. In keeping with the above summary of FcγR binding, complement activation, too, is significantly impaired by the absence of, or a minimal Fc N-glycan (130, 131). *In vitro* binding studies, structural experiments and modeling concur that complement deposition is improved by galactosylated, and reduced by agalactosylated N-glycans (118, 120), with reports on sialylation once more somewhat inconsistent. Since complement activation is dependent upon IgG oligomerization (12), the fact that IgG N-glycan galactosylation aids IgG hexamerization (132, 133) provides a potential mechanism for this observation.

## Is IgG agalactosylation a pro-inflammatory trait?

Taken together, the above observations suggest that immune complexes consisting of autoantigen in combination with agalactosylated (auto)antibodies are less able to activate FcγR- and complement-mediated cellular functions than galactosylated counterparts. Counterintuitively, taken together this suggests agalactosylated IgG may be less able to promote inflammation. Our own work is agreement with this analysis, identifying that immune complexes generated from agalactosylated as opposed to galactosylated IgG are less able to activated pro-inflammatory neutrophil functions (123).

Support for such reasoning also comes from elsewhere. Shortly after vaccinations against tetanus and influenza, antigen-specific IgG1 glycans transiently increase in galactosylation and also sialylation (134). These changes were proposed to support immune responses through presumed anti-inflammatory effects. However, IgG with low fucosylation and high galactosylation generates stronger effector responses against enveloped viruses (135).

Sepsis is characterized by excessive inflammation and immune suppression. In patients with *E.coli*-induced sepsis, IgG glycans contained lower fucosylation in sepsis patients than healthy controls. IgG glycans moreover varied between those patients who survived, and those who did not. Interestingly, non-survivors had less IgG fucosylation and agalactosylation but more mono-and digalactosylation than survivors (136) – conditions that permit better IgG binding to FcγRs. The authors of this intriguing study proposed that agalactosylated IgG glycans may protect surviving sepsis patients from excessive organ damage.

Taking all this into account, and returning once more to autoimmune disease, it stands to reason that the association of agalactosylated IgG glycans with autoimmunity may not be causative. Rather it may be generated as a result of the disease, perhaps as a form of compensatory mechanism aimed at reducing excessive inflammation.
