# Supplementary material for: IgG N-glycosylation in autoimmune diseases
Source: Front Immunol. 2026 Oct 5;17:1933960. doi: 10.3389/fimmu.2026.1933960 (PMC13678527; doi:10.3389/fimmu.2026.1933960)
Supplement: Supplementary file 1 [file DataSheet1.pdf]

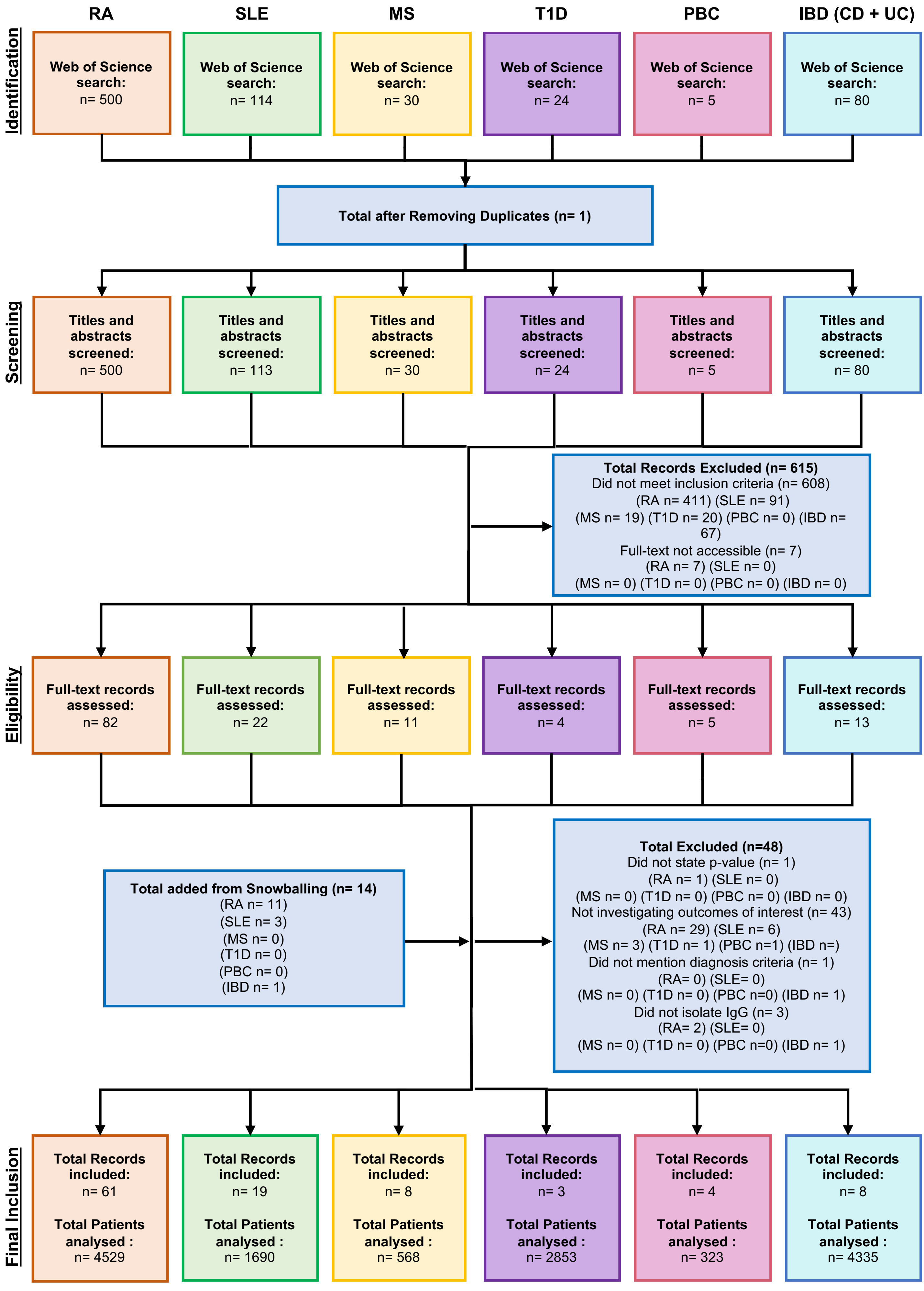

RA
SLE
MS
T1D
PBC
IBD (CD + UC)
Identification
Web of Science search: n= 500
Web of Science search: n= 114
Web of Science search: n= 30
Web of Science search: n= 24
Web of Science search: n= 5
Web of Science search: n= 80
Total after Removing Duplicates (n= 1)
Screening
Titles and abstracts screened: n= 500
Titles and abstracts screened: n= 113
Titles and abstracts screened: n= 30
Titles and abstracts screened: n= 24
Titles and abstracts screened: n= 5
Titles and abstracts screened: n= 80
Total Records Excluded (n= 615)
Did not meet inclusion criteria (n= 608)
(RA n= 411) (SLE n= 91)
(MS n= 19) (T1D n= 20) (PBC n= 0) (IBD n= 67)
Full-text not accessible (n= 7)
(RA n= 7) (SLE n= 0)
(MS n= 0) (T1D n= 0) (PBC n= 0) (IBD n= 0)
Eligibility
Full-text records assessed: n= 82
Full-text records assessed: n= 22
Full-text records assessed: n= 11
Full-text records assessed: n= 4
Full-text records assessed: n= 5
Full-text records assessed: n= 13
Total added from Snowballing (n= 14)
(RA n= 11)
(SLE n= 3)
(MS n= 0)
(T1D n= 0)
(PBC n= 0)
(IBD n= 1)
Total Excluded (n=48)
Did not state p-value (n= 1)
(RA n= 1) (SLE n= 0)
(MS n= 0) (T1D n= 0) (PBC n= 0) (IBD n= 0)
Not investigating outcomes of interest (n= 43)
(RA n= 29) (SLE n= 6)
(MS n= 3) (T1D n= 1) (PBC n=1) (IBD n=)
Did not mention diagnosis criteria (n= 1)
(RA= 0) (SLE= 0)
(MS n= 0) (T1D n= 0) (PBC n=0) (IBD n= 1)
Did not isolate IgG (n= 3)
(RA= 2) (SLE= 0)
(MS n= 0) (T1D n= 0) (PBC n=0) (IBD n= 1)
Final Inclusion
Total Records included: n= 61 Total Patients analysed : n= 4529
Total Records included: n= 19 Total Patients analysed : n= 1690
Total Records included: n= 8 Total Patients analysed : n= 568
Total Records included: n= 3 Total Patients analysed : n= 2853
Total Records included: n= 4 Total Patients analysed : n= 323
Total Records included: n= 8 Total Patients analysed : n= 4335
