# Supplementary material for: IgG N-glycosylation in autoimmune diseases
Source: Front Immunol. 2026 Oct 5;17:1933960. doi: 10.3389/fimmu.2026.1933960 (PMC13678527; doi:10.3389/fimmu.2026.1933960)
Supplement: Supplementary file 2 [file DataSheet2.docx]

Supplementary Material

Caroline Pumpe & Sonja Vermeren, IgG N-glycosylation in autoimmune disease

**A systematic literature search for N-glycans in autoimmune diseases**

Literature searches were conducted on Web of Science and PubMed databases (latest on 16th June 2026) to identify reports on IgG N-glycans associated with autoimmune diseases in 2 steps. Topic searches were TS=((“N-glycan$” OR “glycosylation” OR “glycan$” OR “glycosylates” OR “N-glycosylated”) AND (“IgG” OR “Immunoglobulin G”)) combined with TS=(“rheumatoid arthritis” OR “RA”) *or* TS-(“lupus” OR “SLE” OR “systemic lupus erythematosus”) *or* TS=(“multiple sclerosis”) *or* TS=(”T1D” OR “type 1 diabetes”) *or* TS=(“IDDM” OR “insulin dependent diabetes mellitus”) *or* TS=(“primary biliary cholangitis” OR “PBC” OR “primary biliary cirrhosis”) *or* TS=(“ulcerative colitis” OR “UC” OR “Crohn’s disease” OR “inflammatory bowel disease” OR “IBD”). Hits obtained from PubMed overlapped with those from Web of Science, and duplicates were excluded. Searches were restricted to research papers available in English and published after August 1985. Papers were retrieved for full-text review if their title/abstract contained at least one keywork related to glycosylation and autoimmune disease (see Fig 1). Papers were limited to human studies and included only if (i) association of disease or its treatment with IgG N-glycans was assessed, (ii) the study included suitable controls, (iii) n ≥ 10, (iv) classification of the autoimmune’s disease classification were specified, (v) statistical analysis was performed. Papers reporting hypothetical IgG Fc glycosylation, that had been retracted or that were not accessible as full-text were excluded.

**Fig S1. A systematic literature search for N-glycans in autoimmune diseases.** Literature searches were performed to identify IgG N-glycans associated with autoimmune disease. This was done in two steps, where search 1 identified glycosylation-related papers mentioning IgG and search 2 identified papers on RA, SLE, MS, T1D, PBC and IBD (which comprised CD and UC). Results from combining the two searches were used, with duplicates and papers that did not meet inclusion criteria excluded in two steps as shown. Snowballing identified additional papers citing the first records. Where such papers met the inclusion criteria they were added into the total included records. See supplementary data for details.
